# Early Production of Imperceptible Words by Infants and Toddlers Born Deaf or Blind

**DOI:** 10.1162/opmi_a_00197

**Published:** 2025-04-02

**Authors:** Erin E. Campbell, Charles P. Davis, Martin Zettersten, Molly Cooke, Derek Houston, Naomi Caselli, Elika Bergelson

**Affiliations:** Wheelock College of Education & Human Development, Boston University, Boston, MA; Department of Psychology & Neuroscience, Duke University, Durham, NC; Department of Cognitive Science, UC San Diego, La Jolla, CA; Department of Speech, Language, and Hearing Sciences, University of Connecticut, Storrs, CT; Department of Psychology, Harvard University, Cambridge, MA

**Keywords:** language acquisition, perceptual experience, language access, blindness, deafness, word production, vocabulary

## Abstract

We investigate the roles of linguistic and sensory experience in the early-produced visual, auditory, and abstract words of congenitally-blind toddlers, deaf toddlers, and typically-sighted/hearing peers. We also assess the role of language access by comparing early word production in children learning English or American Sign Language (ASL) from birth, versus at a delay. Using parental report data on child word production from the MacArthur-Bates Communicative Development Inventory, we found evidence that while children produced words referring to imperceptible referents before age 2, such words were less likely to be produced relative to words with perceptible referents. For instance, blind (vs. sighted) children said fewer highly visual words like “blue” or “see”; deaf signing (vs. hearing) children produced fewer auditory signs like hear. Additionally, in spoken English and ASL, children who received delayed language access were less likely to produce words overall. These results demonstrate and begin to quantify how linguistic and sensory access may influence which words young children produce.

## INTRODUCTION

Does being able to see blue or hear a cat’s meow make it easier to learn the words “blue” or “meow”? Children build word knowledge through early sensory, perceptual, motor, and linguistic experiences (Campbell & Bergelson, 2022b; Glenberg & Gallese, 2012; Perniss & Vigliocco, 2014; Smith et al., 2011). More specifically, visual experience with colors, auditory experience with sounds, and linguistic experience with abstract concepts like *tomorrow* might facilitate word learning for visual, auditory, and abstract concepts, respectively. While no one has direct sensory access to abstract concepts, children born blind or deaf also lack access to the range of sensory experiences that are a given for the typically hearing and sighted majority. In what follows, we first consider how sensory experience may be linked to word learning, how this ties to existing empirical data on sighted, hearing, blind, and deaf adults, and how this extends to initial learning in infancy. Against this background, we ask how access to different sources of meaning (i.e., perceptual and linguistic information) may shape the early words children say.

Theories of embodied cognition posit that sensorimotor experience is a requisite part of how humans acquire knowledge and process information (Allport, 1985; Barsalou, 1999). Lending potential support to this, experiments with adults suggest that some sensorimotor simulation occurs during word processing (Davis et al., 2020; Trumpp et al., 2013; Yee et al., 2013). Under the strongest version of these hypotheses, language processing intrinsically involves the activation of prior sensorimotor experiences associated with underlying concepts (Glenberg & Gallese, 2012; Khatin-Zadeh et al., 2021; though c.f., Mahon & Caramazza, 2008). According to these theories, the easier it is to link a word (label) to a sensorimotor experience (referent), the easier that word should be to learn. Indeed, words that refer to objects children can physically perceive or interact with tend to be learned earlier (Muraki et al., 2022; Thill & Twomey, 2016) relative to words that do not correspond with perceptible referents, e.g., “time”, or “think”. If, consistent with embodied cognition, sensorimotor experience is a critical part of acquiring and processing concrete words, we would expect blind individuals’ knowledge of visual words and deaf individuals’ knowledge of auditory words to be compromised^1^. However, evidence from these populations suggests otherwise.

### Sensory Knowledge in Deaf or Blind Adults

Blind and deaf adults possess rich semantic knowledge of perceptual words from imperceptible modalities (Campbell et al., 2024). For example, blind and sighted adult participants produce virtually indistinguishable semantic similarity judgments for sets of perceptual words, including visual words (e.g., “sparkle”, “glimpse”, Bedny et al., 2019), and blind children and adults demonstrate context-appropriate usage of color words and verbs like “look” and “see” both literally (Landau & Gleitman, 1985) and figuratively (Minervino et al., 2018). Blind individuals don’t seem to simply encode visual words as abstract words; rather, blind adults exhibit differentiated neural responses for concepts that are, for them, imperceptible (e.g., “rainbow”) versus concepts that are abstract (e.g., “freedom”) (Striem-Amit et al., 2018). At the same time, neural activity for imperceptible words is also distinct from response patterns for perceptible words (e.g., “rain”), suggesting that blind individuals build rich semantic representations of visual words that differ systematically from the representations of both abstract words and concrete words grounded in direct sensory experience.

Similar results have been found in deaf individuals. For instance, recent work presented native deaf ASL signers with sound stimuli via tactile vibrations (Emmorey et al., in press), and asked them to describe the sounds in ASL. The deaf signers provided signed descriptions for 95% of the sounds, with only 5% receiving “I don’t know” responses. These descriptions included conventionalized signs for auditory properties (e.g., loud^2^), naming sound sources, or using handshapes and movement to visually depict sounds. Relatedly, many sign languages worldwide have signs with highly auditory meanings (e.g., silent, Spread the Sign, 2023). In both blind and deaf populations, therefore, a lack of experience in a given sensory domain does not inhibit acquiring detailed semantic knowledge in that domain, raising doubt about whether sensory experiences are necessary for learning sensory words.

An alternative hypothesis to strong versions of embodied cognition is that semantic knowledge of imperceptible modalities can be derived from language (Campbell & Bergelson, 2022b; Lewis et al., 2019; Saysani et al., 2021; van Paridon et al., 2021). One source of evidence supporting this view is that blind individuals’ semantic associations for color closely resemble those of sighted individuals (e.g., blue–cold, red–hot, Saysani et al., 2021), with word ratings for both groups predicted by the contexts in which color words occur (i.e., words’ distributional semantics in natural language) (van Paridon et al., 2021). In related work testing blind and sighted adults on animal-to-color matching, both groups’ performance was tied to semantic representations derived from natural language (and more strongly so for blind than sighted adults) (Lewis et al., 2019). This further underscores a plausible role for the distributional structure of language in driving semantic representations of perceptual referents (Lewis et al., 2019). In short, prior work suggests blind and deaf adults (like sighted and hearing adults) abstract beyond direct experience, leveraging language input to learn imperceptible words (Campbell & Bergelson, 2022b). How might this ability develop?

### Acquisition of Sensory-Semantic Knowledge

By toddlerhood, sensory systems are relatively mature (Bremner et al., 2012; Leat et al., 2009; Mohan & Dobson, 2000; Schmidt & Beauchamp, 1992; Wild et al., 2017), while language abilities are still rapidly changing. Over toddlerhood, children continue to glean information about words’ meanings from perceptual input, linguistic context, and social cues (Babineau et al., 2022; Bergelson, 2020; Frank et al., 2021). As children are exposed to language across perceptual and linguistic contexts, word representations are refined (Meylan & Bergelson, 2022).

But perceptual input is variably informative for different word meanings, i.e., more so for concrete words, less for abstract ones. For abstract words, learners must rely more on sources like linguistic context (Schwanenflugel & Shoben, 1983) and social cues (Borghi & Binkofski, 2014; Ponari et al., 2020). Thus, words with more perceptually-available referents may be easiest for the youngest language learners (Fourtassi et al., 2019; Schwanenflugel & Shoben, 1983), with children’s abilities to make inferences about abstract word meanings growing as lexical, syntactic, and social understanding increase over developmental time (Bellagamba et al., 2022).

This pattern is reflected in the trajectory of children’s early vocabularies; concrete words tend to be learned before abstract words (Bellagamba et al., 2022; Bergelson & Swingley, 2013; Ponari, Norbury, & Vigliocco, 2018). Seeing, touching, and tasting objects provides rich perceptual information towards developing concept knowledge (Slone et al., 2019; Suarez-Rivera et al., 2022; Yu & Smith, 2012), and words with referents that children can manipulate tend to be learned earlier (Muraki et al., 2022; Thill & Twomey, 2016). In the absence of direct perceptual experience with a word’s referent (which is the case for abstract words for all children, and for visual/auditory words for blind/deaf children, respectively), a central avenue for uncovering that meaning is through linguistic structure, including morphosyntactic cues (Gleitman, 1990) and distributional ones (Vigliocco et al., 2009). The ability to extract this semantic information improves as children develop linguistic competence (Babineau et al., 2022; Bohn et al., 2021).

However, delays in access to structured language input may impede this process. The vast majority of deaf children are born into hearing families where parents’ native language is a spoken language (Mitchell & Karchmer, 2004, 2005). In this scenario, parents’ spoken language input is partially or fully inaccessible to deaf children without use of a hearing aid or cochlear implant. Despite improvements in hearing screenings and auditory technology (Carlyon & Goehring, 2021; Carr & Kihm, 2022; Harrison et al., 2003; Subbiah et al., 2018), even infants identified with hearing loss at birth tend to not receive hearing aids or cochlear implants until months later (Campbell & Bergelson, 2022a; Marnane & Ching, 2015), and these devices do not perfectly restore auditory access (Dettman et al., 2021; Zimmerman-Phillips et al., 2000). If parents instead choose to learn sign language (which is fully perceptually accessible), they must learn it alongside their child, so children’s initial input is not native or fluent (Mitchell & Karchmer, 2005). Regardless of whether language is signed or spoken, recent evidence suggests earlier access (e.g., before 6 months) leads to better language outcomes (Caselli et al., 2021; W. C. Hall et al., 2017; Svirsky et al., 2004; Yoshinaga-Itano et al., 2018).

Given the potential importance of linguistic knowledge for imperceptible words in particular, if vocabulary development is delayed (e.g., due to lack of accessible language input), abstract and imperceptible words may be disproportionately delayed (though, c.f. Ponari, Norbury, Rotaru, et al., 2018). Since early vocabularies are largely composed of highly sensory words, there could be a cyclical effect wherein the lack of foundational linguistic knowledge delays the acquisition of the early vocabulary, which is needed to build further linguistic knowledge (as in the semantic seed hypothesis where a small set of known words scaffold new word learning through syntactic bootstrapping, Babineau et al., 2021; Gutman et al., 2015). The ability to extract semantic information from linguistic structure may thus be disrupted for young blind or deaf learners.

Taken together, the evidence suggests that sensory and linguistic input helps children build rich representations of their early world. For young children born blind or deaf, however, it remains unclear how inaccessible input may influence early learning of perceptible vs. imperceptible words. Our question is not *whether* individuals who are blind or deaf can learn imperceptible and abstract words: it is clear they can (Bedny et al., 2019; Emmorey et al., in press; Landau & Gleitman, 1985; Vigliocco et al., 2018). Rather, we examine whether imperceptible and abstract words are uniquely more challenging to learn based on early perceptual and linguistic experience. In doing so, our study also aims to provide a more precise estimate of the extent to which sensory and linguistic access influence early word production. Towards this aim, we assess early productive vocabulary composition in children who are deaf or blind (alongside hearing/sighted counterparts), and in children who experienced delayed language access (late English or ASL exposure).

### The Present Study: Assessing Early Visual, Auditory, and Abstract Vocabulary

We address two central questions:

1. **To what extent does modality-specific perceptual experience facilitate word production?** We answer this by testing whether congenitally-blind children differ from sighted children in their production of a set of highly visual words, and whether deaf children differ from typically-hearing children in their production of a set of highly auditory words.
2. **To what extent does early access to language input facilitate the acquisition of abstract words?** We answer this by testing whether children first exposed to English or ASL in toddlerhood differ from children with language access from birth in their production of a set of abstract words.

## METHODS

### Instrument

We describe the instrument before the participants, as this will clarify the matching procedure. We measured children’s vocabulary using the MacArthur-Bates Communicative Development Inventory (CDI), a widely used tool for assessing the vocabulary of young children (Frank et al., 2021). The CDI has many versions: we specifically use the American English Words & Sentences CDI for English (Fenson et al., 1994), and the ASL 2.0 CDI for ASL (Caselli et al., 2020), as relevant. The CDI is a parent-report questionnaire assessing children’s comprehension and/or production of a specific set of several hundred words, varying in age-of-acquisition (Fenson et al., 1994). The CDI is a highly valid and reliable instrument (Bates et al., 1994; Fenson et al., 1994), which has been vetted across dozens of languages and developmental contexts and populations (Jarůšková et al., 2023), and reported on in hundreds of published papers (Frank et al., 2021; Marchman & Dale, 2023). While the (American English) CDI is primarily designed for children up to 30 months^3^, we included older children in this study to capture a broader range of language development, particularly among those with language delays. This decision was informed by studies (summarized in the supplemental materials) supporting the validity of its use beyond 30 months in various circumstances (Caselli et al., 2020; Thal et al., 2007).

Notably, even though the CDI is intended to be used holistically, it nevertheless demonstrates good measurement properties, even at the level of individual items, and particularly so for word production (vs. comprehension), used here (Frank et al., 2021, Ch 4.3). Unsurprisingly, given this robustness, past work has demonstrated the utility of the CDI for charting the trajectory of items and word sets for different demographic groups (e.g., Frank et al., 2021, Ch 9). Similarly, recent work has analyzed small subsets of items from the CDI to look at perceptual word learning in particular, with validation of the results confirmed via further analysis of based on naturalistic samples of spontaneous child word production (San Roque et al., 2024).

Moreover, children’s productive vocabulary as measured by the CDI correlates highly (r = 0.65–0.86) with other measures of productive vocabulary, including spontaneous language samples and clinician-administered vocabulary assessments. Notably for present purposes, similarly high CDI validity is found for blind children, deaf children using spoken English (Thal et al., 2007), and deaf children using American Sign Language (Caselli et al., 2020); see Supplementary Information for further validation details and the Discussion section for further considerations regarding use of this instrument and its interpretation. Details regarding word selection for analysis follow the Participants section below.

### Participants and Matching

**Figure 1.**
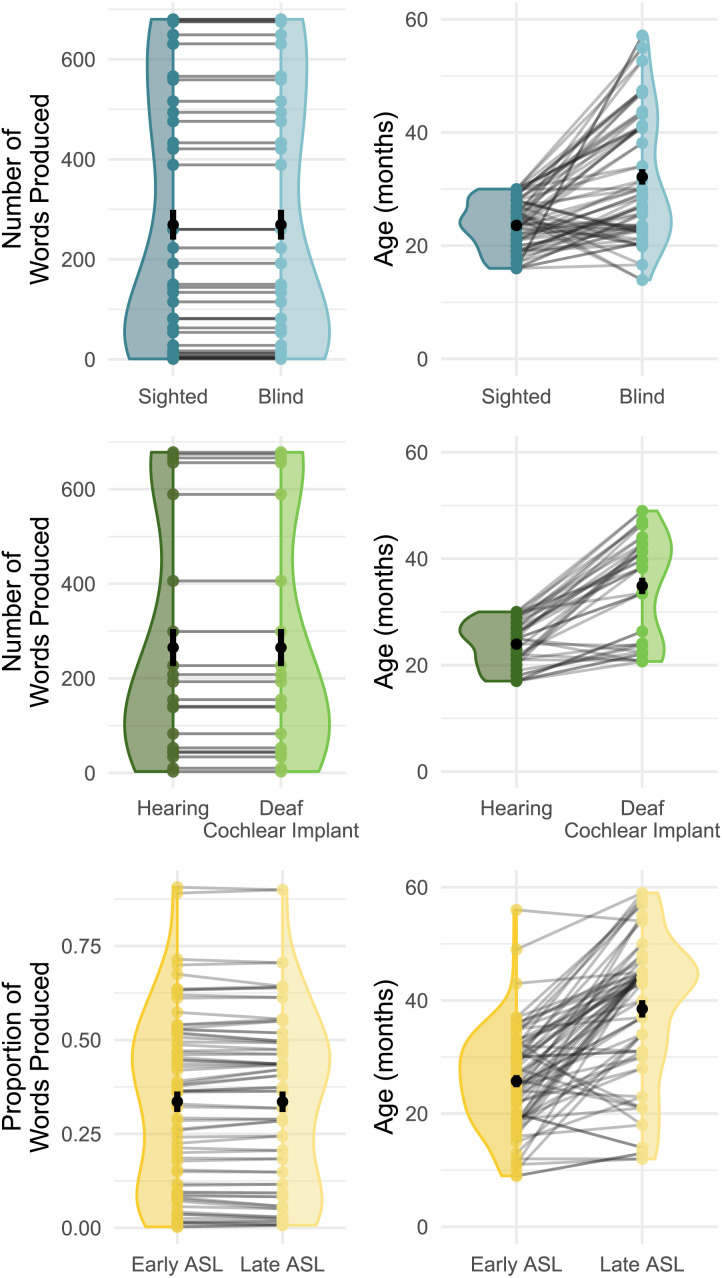
Age and productive vocabulary distributions for each of the samples. Each dot represents the age or vocabulary of one child in the sample. Black point with linerange depicts mean and standard error. Lines connect each participant in the blind, cochlear implant, and late ASL groups to each of their two vocabulary-size matches, see text for details.

Across all six groups of participants, inclusion criteria were: (1) no suspected or diagnosed cognitive or developmental delay, (2) no additional vision or hearing impairment (beyond blindness in the blind group and deafness in the deaf groups), and (3) at least 75% of home language input is in the target language (English or ASL). Each participant in our test groups (blind, deaf spoken language, deaf late ASL) was matched on vocabulary size to *two* participants from our control groups^4^ (respectively: sighted, typically-hearing, and deaf early ASL). This doubling allows us to compare the word production of the difficult-to-recruit test groups to a more precise estimate of word production in the control groups; data for the control groups are pulled from Wordbank, a large database of CDI administrations (Frank et al., 2017). Participant ages and vocabulary sizes are summarized in Figure 1, and demographic information can be found in Table 1.

**Table 1.** Age, vocabulary, and demographic characteristics of each of the groups in our analysis. For continuous variables, range, and mean(SD) are reported. Abbreviations are as follows: U = Unknown. Sex: F = Female, M = Male. Ethnicity: HL = Hispanic or Latino, NHL = Not Hispanic or Latino. Assistive Listening Devices: HA = Hearing Aid, CI = Cochlear Implant, B = Both, N = None.

Group	Age (months)	Language Experience (months)	Productive Vocabulary	Sex	Race	Ethnicity	Assistive Listening Devices
Blind (*N* = 36)	14–57, 32.2 (11.6)	14–57, 32.2 (11.6)	1–680 words, 269 (254) words	F: 50%, M: 50%	Asian: 0%, Black: 3%, Multiracial: 6%, Native American: 3%, Other: 3%, White: 56%, Unknown: 0%	HL: 14%, NHL: 56%, U: 31%	N: 100%
Sighted (*N* = 72)	16–30, 23.6 (4.4)	16–30, 23.6 (4.4)	1–680 words, 269 (252) words	F: 32%, M: 61%, U: 7%	Asian: 1%, Black: 4%, Multiracial: 0%, Native American: 0%, Other: 7%, White: 75%, Unknown: 0%	HL: 4%, NHL: 39%, U: 57%	N: 100%
Deaf - Spoken Language (*N* = 20)	21–49, 34.9 (9.9)	9–39, 21.1 (9)	3–678 words, 265 (251) words	F: 50%, M: 50%	Asian: 0%, Black: 0%, Multiracial: 5%, Native American: 0%, Other: 0%, White: 25%, Unknown: 0%	HL: 0%, NHL: 30%, U: 70%	CI: 100%, N: 0%
Typically-Hearing (*N* = 40)	17–30, 24 (4.3)	17–30, 24 (4.3)	3–678 words, 265 (247) words	F: 35%, M: 55%, U: 10%	Asian: 5%, Black: 10%, Multiracial: 0%, Native American: 0%, Other: 10%, White: 55%, Unknown: 0%	HL: 8%, NHL: 0%, U: 92%	CI: 0%, N: 100%
Deaf - Early ASL (*N* = 72)	9–56, 25.7 (9)	9–56, 25.7 (9)	0–91% of words, 34 (23)% of words	F: 44%, M: 50%, U: 6%	Asian: 0%, Black: 18%, Multiracial: 1%, White: 72%, Unknown: 8%	HL: 8%, NHL: 81%, U: 11%	HA: 12%, CI: 1%, B: 0%, N: 86%, U: 0%
Deaf - Late ASL (*N* = 36)	12–59, 38.5 (13)	4–43, 22.1 (12)	1–90% of words, 34 (23)% of words	F: 56%, M: 44%	Asian: 17%, Black: 6%, Multiracial: 0%, White: 75%, Unknown: 3%	HL: 14%, NHL: 83%, U: 3%	HA: 36%, CI: 17%, B: 33%, N: 11%, U: 3%

As detailed below, because of overall vocabulary size differences across groups, we were able to either match on overall vocabulary size or on age, but not both. Given that we will be zooming into a subset of CDI words (visual, auditory, and abstract) for detailed analysis, our priority was matching the groups we compare on overall vocabulary size and controlling for age by including it as a predictor in all models.

#### Blind and Sighted Samples.

Blind and sighted samples were compared on their vocabulary production reported on the American English CDI (Words & Sentences form). The blind sample consists of *N* = 36 congenitally blind children (13.9–57.1 months, M(SD): 31.88(11.67)), reported by a clinician or caregiver to have “no more than minimal light perception”; these data are described in depth in Campbell et al. (2024). The sighted sample is matched precisely on overall vocabulary size, but given that the CDI is normed for 16–30 months and that most blind children have an overall vocabulary delay relative to sighted peers (Campbell et al., 2024), the vocabulary-size-matched sighted sample (*N* = 72, 16–30 months, M(SD): 23.58(4.43)) is slightly but significantly younger than the blind sample by two-sample Wilcoxon Test (Mean difference = 4.80 months, W = 1,498.00, *p* < .001). Given this, age is included as a predictor in all models. These groups differ in visual access, but not in auditory access or timing of language access.

#### Deaf Spoken Language and Typically-Hearing.

Second, we compare a sample of young cochlear implant users (*N* = 20, 20–49 months, M(SD): 34.74(10.06)) to a Wordbank sample of typically-hearing children (*N* = 40), again matching each deaf participant exactly on vocabulary size to two typically-hearing participants. As above, these groups were compared on the Words & Sentences form of the American English CDI. For the deaf spoken language group, access to both the auditory signal and spoken language was delayed until children received cochlear implants (at 8.03–35.03 months, M(SD): 14.72(6.04)). Following cochlear implant activation, the auditory signal quality tends to be poorer for children with cochlear implants relative to typically-hearing children (Boothroyd & Eran, 1994; Nakisa et al., 2001). As a result, while the groups do not significantly differ in their *length of exposure* to language (Mean difference = 2.50 months, W = 634.00, *p* = .110), the deaf spoken language group was significantly older than the vocabulary-size-matched typically-hearing group (Mean difference = 13.35 months, (W = 1,264.00, *p* < .001); age is included as a predictor in all models. These groups differ in timing and quantity of auditory and language access, but do not differ in visual access.

#### Deaf Early ASL and Deaf Late ASL.

In our third analysis, we compare two groups of deaf children who use ASL: one learning ASL from birth from at least one deaf parent (deaf early ASL) (Caselli et al., 2020) and one group of children learning ASL from non-fluent hearing parents after a period of 6–42 months (deaf late ASL) (Caselli et al., 2021). For the late ASL group, only children whose parents reported using ASL “always” (*n* = 10), “often” (*n* = 14), or “sometimes” (*n* = 12) were included; children whose parents reported using ASL “rarely” or “never” were excluded. After filtering the late ASL dataset to this subsample, we selected a subset of the early ASL group from Caselli et al. (2020) in order to match each late ASL group as closely as possible in age and productive vocabulary^5^ to two early ASL participants. This resulted in a dataset of *N* = 72 early ASL participants (9–56 months, M(SD): 25.74(9.01)) and *N* = 36 late ASL participants (12–59 months, M(SD): 38.50(13.05)); again, these groups differ significantly in age by 2-sample Wilcoxon test (Mean difference = 18.50 months, W = 1,115.00, *p* < .001) but not in length of language exposure (Mean difference = 2.50 months, W = 3,044.00, *p* = .071); age is included as a predictor in all models. Some of the children were reported to use assistive technology, and descriptively, this was more common in the late ASL group (see Table 1). Beyond home experience with caregivers, many participants in our signing samples were also exposed to ASL through intervention services (for hearing parents in particular; e.g., a deaf mentor, early interventionist, or speech-language pathologist), extended family and friends, or ASL pre-school programs or playgroups. These groups differ primarily in timing and quantity of language input, but not auditory or visual input. Caregivers of both groups of children completed the ASL CDI 2 (Caselli et al., 2020); this measure has been validated for use with hearing caregivers who may not be fully fluent in ASL.

It is worth noting at the outset one important sense in which our blind group and our three deaf groups (deaf spoken English, deaf early ASL, and deaf late ASL) are not equivalent. While all children in the blind group have severe to profound vision loss from birth (i.e., at most light perception), the deaf children have a broader range of auditory experience. This is due to wider differences in residual hearing, onset of, and use of assistive technologies. We detail which assistive listening devices were reported by parents in Table 1, and note that while 100% of the deaf spoken English group had CIs, only a small fraction of the early ASL group had any assistive technologies, with the late ASL group falling in between. This is reflective of the wide variability among the deaf population in hearing level and hearing technology outcomes/use (Busch et al., 2017; Campbell & Bergelson, 2022a; Pasta et al., 2021). We cannot more concretely speculate about differences in auditory input without further audiological information (e.g., audiograms, hearing aid/cochlear implant use logs), which is not readily available. These differences in group-level variability in sensory experience are important to bear in mind when we consider the results.

### Word Selection and Lexical Properties

#### English.

For the blind, sighted, deaf spoken language, and typically-hearing groups we analyzed rates of reported production on the American English Words & Sentences CDI for a set of English content words split into 3 sensory modalities: visual (*N* = 10), auditory (*N* = 10), and abstract (*N* = 10); see Table 2.

**Table 2.** Mean lexical properties by referent modality (with standard deviations in parentheses) for the English words (top) and ASL words (bottom) included in our analyses. See Methods text for details and sources of other lexical properties. Words with an asterisk were not rated in the Lancaster Sensorimotor Norms.

Referent Modality	English Words	Frequency	Syllables	Iconicity	Age of Acquisition
abstract	good, bad, time, gentle, finish, wait, love, think, pretend, tomorrow	12,114.10 (14,864.39)	1.50 (0.71)	0.53 (1.13)	27.70 (2.26)
auditory	cockadoodledoo*, grrr*, meow, moo, vroom*, hear, listen, loud, noisy, quiet	1,583.90 (1,880.21)	1.80 (1.23)	2.12 (1.51)	22.80 (5.96)
visual	look, see, black, blue, brown, green, red, white, yellow, dark	15,705.00 (23,170.34)	1.10 (0.32)	0.99 (0.61)	25.30 (1.16)

Referent Modality	ASL Words (English Glosses)	Frequency	Phonological Complexity	Iconicity	Age of Acquisition
abstract	good, bad, time, understand, finish, wait, love, think, pretend, tomorrow	6.31 (0.36)	1.57 (0.79)	3.29 (2.04)	35.43 (5.88)
auditory	deaf, hear, hearing, hearing aid, radio, talk	4.23 (2.84)	1.50 (0.71)	2.05 (0.07)	55.50 (16.26)
visual	black, blue, brown, green, mirror, pink, red, see, white, yellow	5.07 (0.56)	1.33 (0.71)	1.59 (0.98)	28.89 (2.62)

To choose the words for analysis, we used the Lancaster Sensorimotor Norms to seed our initial word selection, ensuring that the chosen words had strong modality-specific perceptual ratings. We then refined this by adding similar words (some of which were not rated in the Lancaster norms) and removing words which could be perceived through other modalities^6^. We further sought to ensure that we had the same number of words for each word-type, for which auditory words proved the limiting factor (there are simply fewer uniquely auditory words on the CDI, and, per the Lancaster Norms, in English in general). This led us to slightly adjust the cutoffs and hand-selection, as detailed below. Specifically:

1. **Visual**: We initially selected words with high Visual ratings (>4.5/5) and relatively low ratings in other perceptual domains (i.e., Perceptual Exclusivity > 0.6). To provide an example, for visual words, this process yielded the following candidates: “butterfly, eye, glasses, lamp, light, picture, backyard, cloud, moon, sky, see, dark, pretty, red, white, yellow.” From this list, we added the remaining color terms based on our assumptions about their inherently visual nature, and removed words that could plausibly be experienced through other modalities (e.g., “backyard,” “glasses,” “eye”).
2. **Auditory Words:** We initially selected words with high Auditory ratings (> 4/5) and relatively low ratings in other perceptual domains (i.e., Perceptual Exclusivity > 0.6). This yielded the following candidates: “quiet”, “call”, “loud”, “hear”, “meow”, “noisy”, “listen”, “moo”. From this list, we added the remaining onomatopoeias, which were not included in the Lancaster Norms (again, based on our assumption that these too are inherently auditory-related).
3. **Abstract Words**: Abstract words were chosen based on their low perceptual ratings across all domains (i.e., < 2.5/5 for all senses) and lack of domain specificity. To align these better with the types of words in the visual and auditory sets, we imposed the additional constraint that these words couldn’t be function words (based on CDI’s lexical class categorization). From this list, we included several of the time-related words (“time,” “tomorrow”), some words about emotion or cognition (“think,” “pretend,” “love”), and some about states of completion or delay (“finish”, “wait”).

In order to control for relevant word-level properties statistically, we also computed frequency, phonological complexity, and iconicity. These lexical co-variates were selected to control for differences across referent modalities that are not germane to our research questions, e.g., that visual words are more frequent, and auditory words (in spoken language) more iconic. Moreover, these covariates represent three of the most robust and cross-linguistically consistent predictors of age of acquisition (Braginsky et al., 2019; Perry et al., 2015). Frequency was calculated using the childesdb package (Sanchez et al., 2019) based on how often children heard our 30 words in the North American English corpora in CHILDES, a database of child-centered language (MacWhinney, 2014). Word frequency was then converted to a log scale given its Zipfian distribution (Lavi-Rotbain & Arnon, 2023). As a proxy for phonological complexity (i.e., how challenging the word is to produce), we also include number of syllables in the citation form of the word. Iconicity ratings come from Winter et al. (2017), wherein participants rated words on a scale from −5 (word sounds like the opposite of its meaning; not iconic at all) to 5 (word sounds like what it means; highly iconic).

#### ASL.

For the Early and Late ASL groups, we selected visual (*N* = 10), auditory (*N* = 6), and abstract (*N* = 10) words from the ASL CDI 2.0 by polling deaf and hearing adult signers of ASL on which words were most strongly associated with each of the categories. As might be expected, the ASL CDI contains largely overlapping visual and abstract words, but fewer exclusively “auditory” words than the American English CDI. When possible, we selected English/ASL words translation equivalents of each other; this was the case for 9/10 abstract words (*gentle* was only included in the English analyses; understand was only included for ASL) and 8/10 visual words (*look* and *dark* were only included in the English analyses; mirror and pink were only included for ASL), and 1 auditory word (*hear*/hear); the full list of English and ASL auditory words and all other items we analyzed are provided in Table 2.

**Figure 2.**
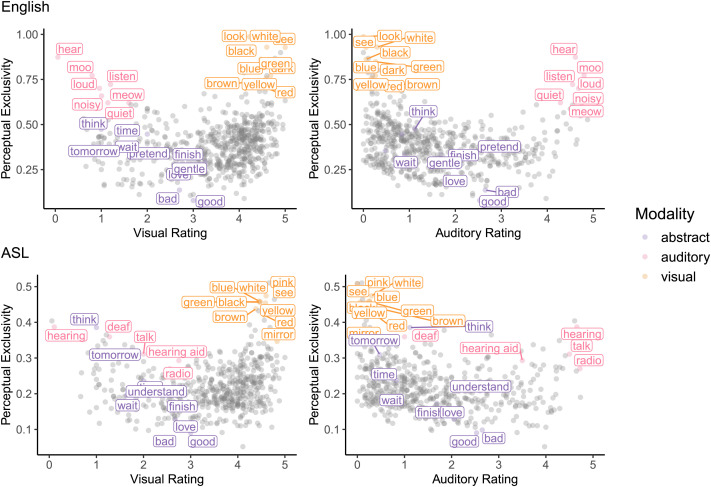
CDI words plotted by Visual Rating and Perceptual Exclusivity (left), and Auditory Rating and Perceptual Exclusivity (right). Selected words are labelled and colored by referent modality (visual, auditory, or abstract). Top row: American English Words and Sentences form. Higher perceptual ratings (y axis) reflect more exclusivity to one perceptual domain; lower ratings reflect greater multimodality. The auditory words cockadoodledoo, vroom, and grrr are omitted from this figure because they are not rated in the Lancaster Sensorimotor Norms. Bottom row: ASL CDI 2.0, plotted by perceptual rating for their English gloss.

To control for the relevant word-level properties, we used lexical ratings from ASL-LEX (Caselli et al., 2017; Sehyr et al., 2021); see Table 2 and Figure 2. To summarize these lexical properties briefly, frequency ratings^7^ were produced by a sample of deaf signers rating how often a sign appears in everyday conversation on a scale from 1 (*very infrequently*) to 7 (*very frequently*) (Caselli et al., 2017). Phonological complexity refers to the number of complex features the sign has (Morgan et al., 2019); this score can range from 0 (no complex features) to 7 (contains all seven features). Like the syllables measure for spoken language, signs that are more phonologically complex tend to be longer in duration, less frequent, and acquired later (Caselli & Pyers, 2017, 2020; Sehyr et al., 2021). For iconicity, we used ratings from deaf native signers when available; deaf signers were asked to rate signs on a scale from 1 (*not iconic at all*) to 7 (*highly iconic*). For five of the words (white, deaf, pink, tomorrow, hearing), deaf native signer ratings were unavailable, and ratings from hearing non-signers for the signs (using the same rating procedure) were substituted (Caselli et al., 2017); iconicity ratings from signers and non-signers tend to be strongly correlated with each other (Caselli & Pyers, 2017; Sehyr et al., 2021).

### Data and Code

All analyses were conducted in R, using primarily the lme4, emmeans, and ggggeffects packages. Data and code for all analyses are available on OSF, as are full citations for all R libraries used. These analyses were not preregistered.

## RESULTS

For each of three comparisons (blind/sighted, deaf spoken language/typically-hearing, or deaf early ASL/deaf late ASL), we constructed a series of mixed effect logistic regression models predicting the probability of word production, given child characteristics and lexical properties. Specifically, each model estimates the likelihood of word production based on child age, several word-level predictors (frequency, phonological complexity, and iconicity), group (blind vs. sighted, deaf spoken language vs. typically-hearing, or deaf early ASL vs. deaf late ASL depending on the model), referent modality (abstract, auditory, or visual), and an interaction between group and referent modality. This interaction was our primary effect of interest, as its presence would indicate that the likelihood of word production varies differentially across auditory, visual, or abstract words as a function of linguistic and sensory access. In all models, we also included a random intercept for participant, to account for multiple observations (words) from each child. For transparency, we intended to include a random intercept for word, but the models with a random intercept for word did not converge, except where noted below.

### Blind Children and Sighted Peers

We first compared production of our 30 visual, auditory, and abstract words in two groups of children that differ in visual but not auditory or language access: blind toddlers and sighted peers matched on overall vocabulary size per the American English CDI. Our model formula is: **Production ~ Age + Group**_**Blind vs. Sighted**_ * Referent Modality_**Abstract vs. Auditory vs. Visual**_ **+ log(Frequency) + Syllables + Iconicity + (1| Participant)**.

**Figure 3.**
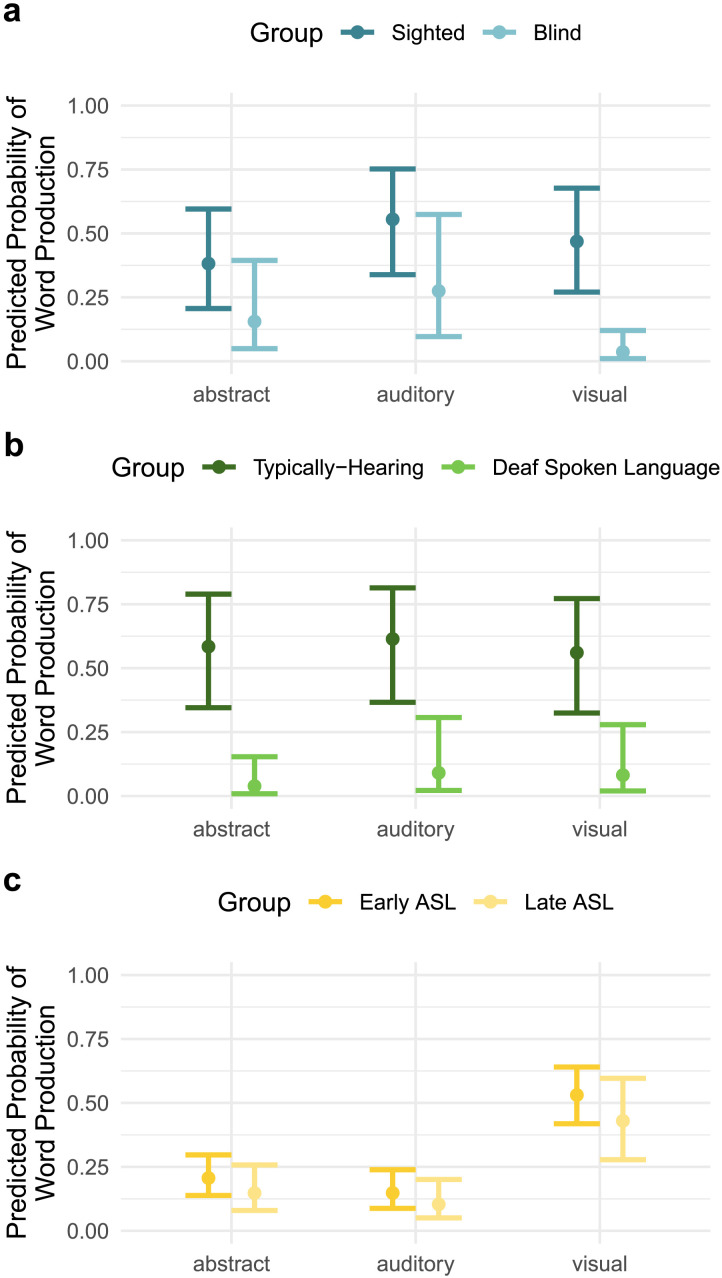
Predicted probabilities of word production by referent modality and group, controlling for age, frequency, phonological complexity, and iconicity; see text for model formulas. A. Blind and sighted participants. B. Deaf spoken language and typically-hearing participants. C. Deaf early ASL and Deaf late ASL participants. Whiskers show CIs around the mean. N.b., predicted probability values should not be compared across panels. Vocabulary size was not a predictor but rather controlled for within each analysis by matching individuals across groups on overall vocabulary size.

As expected, we found that these 30 words were significantly more likely to be produced by older children, and when they were shorter and more iconic; there was no effect of word frequency among these already high-frequency words. We further found that while blind and sighted children were equally likely to produce these words overall (i.e., no main effect of group), there was a significant main effect of referent modality such that across groups, abstract words are 50.4% less likely to be produced than auditory words. Most notably, we found a significant interaction between group and referent modality, such that sighted children were significantly more likely to produce visual words than blind children, but did not differ in their production of auditory or abstract words. This effect on visual words was large: sighted children were 7.08 times more likely than blind children to produce highly visual words (Figure 3A). See Table 3 for full model results and reference level details.

**Table 3.** Model estimates for blind and sighted participants. In this model, Sighted is the reference level for Group, and Auditory is the reference level for Referent Modality.

Variable	Beta (log odds)	Standard Error	*p*
Age (months)	0.25	0.05	<.001
log(Frequency)	−0.04	0.05	.364
Syllables	−0.84	0.09	<.001
Iconicity	0.77	0.07	<.001
Referent Modality (abstract)	−0.70	0.23	.002
Referent Modality (visual)	−0.35	0.21	.100
Group (blind)	−1.19	0.81	.144
Modality(abstract): Group	−0.02	0.34	.947
Modality(visual): Group	−1.96	0.35	<.001

### Deaf Children Learning Spoken Language and Typically-Hearing Peers

We next compared children that differ in auditory and language access, but not in visual access: Deaf children learning spoken language and their typically-hearing peers matched on overall vocabulary size per the American English CDI. Our model formula is: **Production ~ Age + Group**_**Deaf Spoken Language vs. Typically-Hearing**_ * Referent Modality_**Abstract vs. Auditory vs. Visual**_ **+ log(Frequency) + Syllables + Iconicity + (1| Participant)**.

Here too, words were significantly more likely to be produced by older children, and when they were more shorter and more iconic; again we find no frequency effect for this set of words. In this model, we found a significant main effect of group: overall, typically-hearing children were 14.35 times more likely to produce one of these words than deaf children learning spoken language. For the deaf spoken language group (but not hearing peers) we found significant differences between referent modalities: the difference between groups was greater for abstract words, relative to visual words. That is, typically-hearing children were 2.43 times more likely than their deaf peers to produce abstract words; see Figure 3B. While the typically-hearing group was also significantly more likely than the deaf spoken language group to produce auditory words, the size of this difference was not significantly greater than the difference between groups for visual words (which were the reference level for comparisons). See Table 4 for full model results and reference level details.

**Table 4.** Model estimates for deaf (cochlear implant) and typically-hearing participants. In this model, Hearing is the reference level for Group, and Visual is the reference level for Referent Modality.

Variable	Beta (log odds)	Standard Error	*p*
Age (months)	0.29	0.05	<.001
log(Frequency)	−0.03	0.06	.555
Syllables	−0.90	0.12	<.001
Iconicity	0.93	0.08	<.001
Referent Modality (abstract)	0.10	0.25	.696
Referent Modality (auditory)	0.22	0.27	.413
Group (deaf)	−2.66	0.98	.006
Modality(abstract): Group	−0.89	0.42	.034
Modality(auditory): Group	−0.11	0.38	.778

### Deaf Children with Early ASL Access and Deaf Children with Late ASL Access

Lastly, we compared two groups of children learning ASL who were matched on overall vocabulary size per the ASL CDI: one exposed to language from birth and one after a delay. Here, the two groups differ in language access but not in visual or auditory access. Our model formula is: **Production ~ Age + Group**_**Deaf Early ASL vs. Deaf Late ASL**_ * Referent Modality_**Abstract vs. Auditory vs. Visual**_ **+ Frequency + Phonological Complexity + Iconicity + (1| Participant)**.

Here again, older children were more likely to produce a given word. Words that native signers rated as more frequent and less phonologically complex were more likely to be produced; there was no effect of iconicity. We also did not find a significant effect of language access (i.e., no main effect of group): early vs. late ASL groups did not significantly differ in their likelihood of sign production for this set of words. While visual signs were 4.79 times more likely to be produced than abstract signs and 6.39 times more likely to be produced than auditory signs, these differences did not vary significantly by group. Finally, unlike in the comparisons above, there was no interaction between group and word type. See Figure 2.C and Table 5.

**Table 5.** Model estimates for early ASL and late ASL participants. In this model, early ASL is the reference level for Group, and Visual is the reference level for Referent Modality.

Variable	Beta (log odds)	Standard Error	*p*
Age (months)	0.10	0.02	<.001
Frequency Rating	0.36	0.09	<.001
Phonological Complexity	0.28	0.09	.002
Iconicity	−0.02	0.04	.634
Referent Modality (abstract)	−1.57	0.21	<.001
Referent Modality (auditory)	−1.86	0.29	<.001
Group (Late ASL)	−0.52	0.44	.239
Modality(abstract): Group	0.29	0.27	.294
Modality(auditory): Group	−0.04	0.48	.935

### Exploring the Interaction Between Language Access and Perceptibility on Early Word Production

While children who received delayed access to *spoken language* were especially less likely to produce abstract words (relative to visual words); children who received late access to ASL didn’t show an analogous effect. To better understand this discrepancy, we conducted an additional exploratory analysis designed to maximize the possibility of finding an effect of language access. To increase statistical power, we combined the groups from the second and third analysis into an *early language input group* (typically-hearing children and deaf children learning ASL; all have full language access from birth) and a *late language input group* (deaf children with cochlear implants learning spoken English and deaf children learning ASL at a delay; see Table 1). This gave us a larger sample size (n_Early Language Access_ = 112, n_Late Language Access_ = 56), while maintaining the vocabulary-size matching. The subgroups within each language access group do not significantly differ from each other in age or amount of language experience (all *p*s > .05 by *t*-test).

Since children in these groups were variable in auditory input and assistive technology use as noted above, this analysis focuses on words that were equally perceptible or imperceptible to all children in this subsample: visual and abstract words. Since the lexical properties differ across spoken English and ASL (log corpus-based frequency for English vs. native speaker frequency ratings for ASL; syllable length for English vs. phonological complexity for ASL), we z-scored words’ frequency, phonological complexity, and iconicity separately by language and then used these z-scored values as the lexical properties in the model. The larger sample size in this analysis allowed us to include a more complex random effects structure. In addition to the random intercept for participant, we included a random intercept for word, nested within language: Production ~ Age + Frequency + Phonological Complexity + Iconicity + Language Timing * Referent Modality + (1| Participant) + (1| Language/Word); see Table 6. Due to the structure of the underlying data (i.e., vocabulary-size matching within but not across languages), this analysis does not make any claims about the relative merits of either communication approach, just on the timing of children’s access to language.

**Table 6.** Model estimates for early language access and late language access participants. In this model, Early Access is the reference level for Group, and Visual is the reference level for Referent Modality.

Variable	Beta (log odds)	Standard Error	*p*
Age (months)	0.19	0.02	<.001
Frequency z-score	0.44	0.31	.152
Phon. Complexity z-score	0.02	0.21	.935
Iconicity z-score	0.06	0.17	.750
Referent Modality (abstract)	−1.31	0.41	.001
Group (Late Access)	−1.86	0.54	<.001
Referent Modality: Group	−0.06	0.25	.814

**Figure 4.**
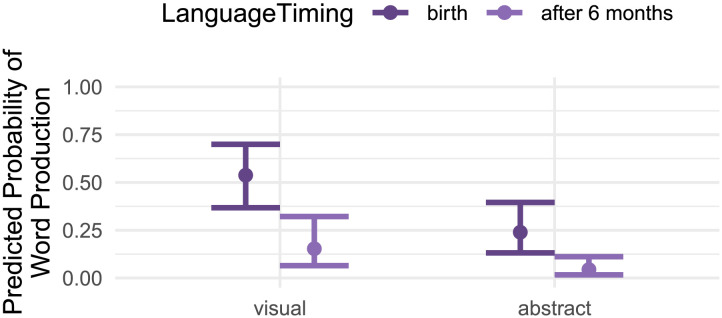
Predicted probabilities of word production by language timing and referent modality, controlling for age, frequency, phonological complexity, and iconicity.

Children who received early access to language input (i.e., beginning between birth and six months) were 6.42 times more likely to produce words, and visual words were 3.69 times more likely to be produced than abstract words. Critically, however, there was no significant interaction: early versus late access to language did not differentially affect the production of visual vs. abstract words; see Figure 4.

## DISCUSSION

Our first research question was whether modality-specific perceptual experience facilitates word production. This can be answered affirmatively: across our range of participants with varying visual, auditory, and linguistic access, children were less likely to produce words in imperceptible domains, relative to perceptible domains. This extends prior work about the acquisition of concrete vs. abstract words (Bellagamba et al., 2022; Bergelson & Swingley, 2013; Della Rosa et al., 2010; Lux et al., 2021; Pexman, 2019; Ponari, Norbury, & Vigliocco, 2018), suggesting that it is not whether referents are concrete *per se* that drives their acquisition, but the extent to which children themselves can perceive them directly through the senses. Additionally, our findings not only reinforce the intuitive expectation that children are less likely to produce words referring to imperceptible referents, but also provide a more precise estimate of the magnitude of this difference across a variety of developmental contexts. Our second research question asked whether early access to language input facilitates the acquisition of abstract words in particular. Here our results were more mixed: delays in language exposure (spoken or signed) were linked with delayed word production overall, but less clearly so for abstract words in particular. We discuss the implications of these results in turn.

In our first comparison, controlling for age and lexical properties, we found that young blind children and their sighted peers matched on overall productive vocabulary size did not differ in their likelihood of producing words that were equally perceptible (i.e., our auditory words) or *im*perceptible (i.e., our abstract words). Blind children were, however, substantially less likely to produce the highly visual words we tested. This converges with prior work considering the composition of blind vs. sighted children’s overall productive vocabulary, rather than just the most strongly visual words considered here (Campbell et al., 2024). That work largely finds no difference in the types of words each vocabulary-size matched blind and sighted children produce across many dimensions (e.g., syntactic class, topic), except that blind children are more likely to produce visual words that can be experienced through other perceptual modalities, relative to words exclusively experienced through vision. The current results add precision to this previous holistic full-vocabulary analysis.

Notably, even early in childhood, when these highly visual words enter the lexicon, blind children have rich semantic representations of their meanings. As Landau and Gleitman (Landau & Gleitman, 1985) reported, *“By age 36 months, [a blind child] was using these terms freely and frequently in conversation, in ways that seemed appropriate–except we knew she was blind”* (pg. 52). This landmark work also showed that their subject Kelli had detailed semantic knowledge of perception verbs and colors words (e.g., knowing ideas don’t have colors). Later work replicated and extended this finding of semantic sophistication in older children and adults (Bedny et al., 2019; Minervino et al., 2018; Striem-Amit et al., 2018). Our work does not refute this literature; rather, it deepens the empirical knowledge base by showing that the composition of the early lexicon differs in modality-specific ways, a point to which we later return.

In our second comparison, deaf children learning spoken language were significantly less likely to produce the words we queried (across all modalities), compared to hearing peers who had the same overall vocabulary size. This suggests that the differences in deaf and hearing children’s experiences seem to be changing the early spoken vocabulary more broadly–across all of the word types studied–rather than just the auditory words, which were the focus of our earlier hypothesis. This could be interpreted as a spoken language vocabulary delay for deaf children, which is consistent with prior work (Campbell & Bergelson, 2022a; González Cuenca et al., 2020; Takahashi et al., 2017; Yoshinaga-Itano et al., 2017), and may indicate a more general effect of delayed linguistic access on deaf children’s spoken vocabulary. Strikingly, the spoken vocabulary delays among deaf children were notably large for abstract words. This may be because abstract word meanings are particularly heavily scaffolded by language, so language delays may affect them disproportionately (Vigliocco et al., 2009). Our results here also converge with prior work (Jung et al., 2020) that finds older children with cochlear implants use more concrete nouns and fewer onomatopoeia and social words than their younger typically-hearing peers. However, it remains challenging to separate effects of timing vs. acoustic fidelity of auditory and linguistic input, as the deaf spoken language and typically-hearing groups in our study differ from each other in all of these regards.

We approached this issue more directly in our third comparison by measuring vocabulary acquisition in children with similar auditory input but differences in the timing and fluency of language input. Here, we compared deaf children learning ASL from birth from deaf parents, and deaf children receiving ASL from hearing parents after 6–42 months. If early access to language input differentially supports abstract words, we would have found that, like the spoken language comparison above, abstract words would be disproportionately absent in the vocabularies of children with late access to sign. Instead, we found that both ASL groups showed the same pattern: while children were more likely to produce visual words overall, there were no modality-specific differences in the likelihood of producing abstract, auditory, or visual words between the early and late ASL groups. This suggests early ASL access does not differentially support abstract words, at least as measured here.

Lastly, to further query the influence of language access on abstract word production, we combined two early language groups (typically-hearing and deaf early ASL) and two late language groups (deaf spoken language and deaf late ASL). We found that delays in language access (independent of language modality) resulted in delays in language production. Consistent with prior research (Bellagamba et al., 2022; Bergelson & Swingley, 2013; Ponari, Norbury, & Vigliocco, 2018), we also found that abstract words were less likely to be produced than more concrete (in this case, visual) words. However, we did not find evidence that language access predicted production differentially for visual and abstract words.

Overall, our findings support the uncontroversial idea that domain-specific perceptual experience with a word’s referent influences likelihood of production, through the lens of two types of sensory impairment. Our blind group was significantly less likely to produce visual words than our typically-sighted group, and our deaf group was less likely to produce auditory words than our typically-hearing group (though they were also less likely to produce visual words).

We caution against the interpretation that the differences we observe reflect deficits for deaf or blind children. Rather, children may adaptively deprioritize words referring to imperceptible referents in favor of words that are more useful to their everyday experiences. These results do not suggest that children born deaf or blind are incapable of producing words about sound or sight; in our sample, blind children as young as 16 months were producing color words, and deaf children as young as 18 months were producing signs like hear. Given that these types of word meanings are perceptually unavailable or less available to these children, the results rule out a strictly embodied account of early word learning and instead support the idea that while perceptual experience facilitates word learning, especially in early childhood (Muraki et al., 2022; Thill & Twomey, 2016), language input itself plays a strong role in relaying meaning regardless of sensory access (Gleitman, 1990; Gleitman & Newport, 1995; Landau & Gleitman, 1985).

Turning to language access, our results suggest that accessible language input is important for word learning. That said, early accessible language input does not seem to disproportionately support abstract words vs. perceptible words, though this may be influenced by language modality (i.e., spoken vs. signed). Deaf children learning spoken language through cochlear implants (i.e., no language access prior to implantation at 8–35 months) had overall lower vocabulary production relative to hearing peers, but were especially less likely to produce abstract (vs. visual) words. This was in contrast to blind children (who have full access to spoken language from birth): Blind children did not produce fewer of these 30 words overall, but, as expected and like their sighted matches, produced fewer abstract words than auditory words. We also did not observe the same pattern in our ASL groups (ASL exposure from birth vs. delayed ASL access (i.e., no language access for 6–42 months)). Here we saw lower rates of abstract and auditory word production (relative to visual words) but no difference between Early vs. Late ASL access. In contrast, combining spoken and ASL groups by early vs. late language access in our exploratory analysis, we found that visual words were more likely to be produced than abstract ones, as expected, but also that later language access led to lower production overall. But critically, word type (abstract vs. visual) and access (late vs. early) did not interact. That is, even with increased statistical power, the timing of language access did not disproportionately affect abstract words (though this pattern of results merits replication, given its exploratory nature). Another possibility for the null interaction with abstract words is that we are observing a floor effect: The children with late language access are producing few abstract words at all, and if late language access does have an outsized effect on abstract words, we may need to study older children in order to observe that interaction.

It is also worth noting the potential variability in auditory input among the cochlear implant group and the ASL groups. Such differences could stem from differential residual hearing, frequency of wearing hearing aids or cochlear implants, and the audiological efficacy of assistive devices. If experiences with sound matter for children’s production of auditory words, we might expect large within-group variability. That is, children with minimal auditory input may exhibit floor effects for auditory word production, while those with extensive and effective CI use could approach typically-hearing performance. This variability could mask potential differences between groups. Evaluating such possibilities awaits larger-scale samples with more detailed information regarding auditory input along dimensions like those mentioned above.

### Limitations and Avenues for Future Research

Our approach implicitly assumes that language exposure and perceptual experience are independent influences, but it is plausible that they are interrelated. Although parents don’t seem to talk less to children with sensory impairments (Campbell et al., 2021; Dirks et al., 2020; VanDam et al., 2012), there is evidence that more fine-grained aspects of language input to children with sensory impairments may differ from language input to their typically-sighted/hearing peers (Ambrose et al., 2015; Andersen et al., 1993; Dirks et al., 2020; Pérez-Pereira & Conti-Ramsden, 2001). For example, prior research suggests that parents of typically-sighted/hearing children tailor language input to things their children are presently attending to (Tamis-LeMonda et al., 2013). If deaf children attend less to auditory stimuli and blind children attend less to visual stimuli, then it stands to reason that parents may talk less about these properties. Recent work from Campbell et al. (2023) suggests that parents of blind children use visual words at similar rates to parents of sighted children, but it’s possible that the social and linguistic contexts in which these particular words are used could vary across groups in ways that could influence words’ learnability. Testing this possible explanation further awaits measures of naturalistic interactions between children with differential sensory access and their caregivers.

Perhaps relatedly, some of the children in the ASL groups may have also been learning English (though all reported that ASL was their dominant language), and we unfortunately do not have information on their English language skills. This raises interesting questions about which language(s) a word is likely to be learned in, and whether this varies based on a words’ perceptual-semantic content: Among ASL-English bilinguals, would words pertaining to auditory experience be likely to appear only in children’s English vocabulary? Such an effect could arise due to input properties of the word in the respective languages, or perhaps due to other lexical properties (e.g., iconicity) which could influence the relative difficulty of learning a word in ASL vs. English. We leave this intriguing question to future research.

It’s also worth considering how parents might approach the vocabulary instrument differently based on their child’s sensory abilities: what does it mean when a parent reports that their child says a word? In part, the task is an exercise in parent’s episodic memory, whether they recall specific instances of their child producing a given word (Frank et al., 2021). Simultaneously, parents may be influenced by their perception of a given word’s difficulty relative to specific words they *do* recall their child saying. This could be biased by their knowledge of a specific child’s interests (e.g., *“She loves vehicles. She definitely says all of those words.”*) or abilities (e.g., *“If my child is blind, he couldn’t be saying ‘blue’, right?”*), such that sensory words may be systematically under-reported for blind or deaf children. On the other hand, parents of children born blind or deaf are often extremely sensitive to the particulars of their child’s development and sensory experience, and we suspect that their production of these “imperceptible” words would be highly salient in parents’ memory. In either case, by measuring the patterns of groups (with Ns larger than typically used in such work, particularly for blind children), we hope to mitigate any idiosyncratic response tendencies of individual parents. That said, parental report instruments always have an increased risk of reporting bias relative to direct measures (which have their own pros and cons).

In an effort to address this concern more quantitatively, we provide new validation data and summarize prior validation efforts as Supplemental Information. Encouragingly, across a wide range of validation approaches, there is no evidence that parents of deaf children learning spoken or signed language, parents of blind children, or parents of hearing/sighted children differ notably in how well the CDI correlates with other measures of child language (spontaneous samples, other instruments, etc.); correlations across the board range from .6 to .9. This mitigates concerns regarding the parent report nature of the current results. It nevertheless remains possible that parents of children with different sensory abilities report their children’s production of the words we analyze here differently from each other. This is an intrinsic limitation of our use of the CDI to query a specific set of a few dozen words across populations with different sensory access. Regarding those few dozen words, our analysis focused on a unique pocket of words referring to concepts that are experienced exclusively or primarily through vision or hearing, and as such, it does not represent children’s overall vocabulary—nor was it intended to. Notably, this approach led to an overrepresentation of color words in the visual list and onomatopoeias in the English auditory list, reflecting the perceptual-semantic constraints we aimed to investigate. We look forward to future work looking at spontaneous production which will let us more thoroughly tackle differences in production of different perceptual words beyond those on the CDI.

A further limitation of our work is that we looked at age at first language exposure, rather than cumulative language exposure, which may be a more sensitive measure. Capturing a more holistic picture of children’s language exposure (e.g., audibility of spoken language, effectiveness and frequency of hearing aid/cochlear implant use, frequency of ASL exposure, fluency of parent in ASL, M. L. Hall, 2020; M. L. Hall & De Anda, 2022) could help determine how and when the language signal is informative for acquiring the meaning of words, especially abstract ones. In the deaf population in particular, adopting a more nuanced measurement of language input could help reconcile differences in our results between children learning spoken language and children learning sign language. As noted above, the deaf and blind groups in our study are not equatable in a simple way as the former has a wider range of auditory access; we nevertheless find that considering the results of these groups side by side stands to inform our understanding of the roles of perceptibility and language access within word learning.

Lastly, we analyzed early word production, which is generally well-preceded by early receptive knowledge (e.g., Bergelson & Swingley, 2012). Understanding the latter will be an important complement to the present work. For instance, early literature labelled production of imperceptible words by blind individuals as “verbalisms”, assuming that missing perceptual access rendered their semantic representations meaningless (Cutsforth, 1932). Based on data from older children and adults showing nuanced semantic knowledge of words with imperceptible referents (Bedny et al., 2019), it’s now clear that this is an ableist mischaracterization of the production data in deaf and blind children. While measuring receptive semantic knowledge of imperceptible words is beyond the scope of this work, we look forward to further research aimed at characterizing such representations in young children with varying early life experiences.

### Conclusion

Across three sets of analyses targeting the early vocabularies of children who vary in access to visual, auditory, and linguistic information, young children were less likely to produce words that refer to an inaccessible referent, in both predicted and unexpected ways. Firstly, blind children were less likely to produce the highly visual words we analyzed than their sighted peers were, but not our abstract or auditory words. Critically, this group difference was limited to the visual words: both sighted and blind infants produced the abstract words less than auditory words overall. Secondly, deaf children learning spoken language were less likely to produce auditory words than their hearing peers. However, they were also less likely than hearing peers to produce our visual words and especially our abstract words. Finally, deaf children learning sign language were less likely to produce auditory words (and abstract words) than visual words – i.e., both words that were less perceptible to all children (abstract words) and uniquely less perceptible to them (auditory words). The role of language access in scaffolding acquisition of imperceptible meanings was less straightforward, but our exploratory analysis suggests that late access to language affects both perceptible and abstract words. Taken together, our findings build on a longstanding literature highlighting clear links between children’s experiences and their emerging vocabularies. We add that these links are shaped by access to information about the world obtained both through language and the perceptual system, across wide-ranging and divergent early sensory and linguistic experiences.

## FUNDING INFORMATION

NSF GRFP (2019274952) to Erin Campbell. NIDCD R01DC008581 to Derek Houston. NIDCD R01 DC018279 and R21 DC016104 to Naomi Caselli. NSF CAREER BCS-1844710 to Elika Bergelson.

## AUTHOR CONTRIBUTIONS

E.E.C.: Conceptualization; Data curation; Formal analysis; Investigation; Visualization; Writing – original draft; Writing – review & editing. C.P.D.: Validation; Writing – original draft; Writing – review & editing. M.Z.: Conceptualization; Writing – review & editing. M.C.: Project administration. D.H.: Resources; Writing – review & editing. N.C.: Resources; Writing – review & editing. E.B.: Conceptualization; Resources; Supervision; Writing – review & editing.

## DATA AVAILABILITY STATEMENT

Data and code for all analyses are available on OSF.

## Notes

^1^ We use “{visual, auditory, abstract} words” as shorthand for words that refer to visual, auditory, or abstract concepts. Also, as admittedly imprecise shorthand, we refer to visual and auditory words as “imperceptible” to blind and deaf participants, respectively (by dint of their sensory difference in the predominantly relevant sense), and abstract words as “imperceptible” to all participants. As clarified later, this coarse-grained classification cannot fully capture individuals’ sensory experiences with “imperceptible” words for a variety of reasons (e.g., a deaf child experiencing “loud” via tactile vibrations, residual hearing, etc.).^2^ English labels of ASL signs are indicated with small caps.^3^ The ASL CDI is designed for children up to 60 months (Caselli et al., 2020).^4^ All test participants could be matched to two or more controls while maintaining vocabulary-size matching; we opted for a consistent 2 matches each, rather than letting this vary across participants. When more than two control participants met the matching criteria, the first two participants by Wordbank’s DataID column were selected. Results are robust to whether we match to one control participant or two, and to whether we select the first two matches or the last two matches (by WordBank DataID).^5^ Unlike the other groups, ASL groups’ vocabulary matching was based on the *proportion* of produced words out of the number of words that parents responded to, given that the ASL CDI provides an option to “skip” words (Caselli et al., 2020). While some English CDI administrations permit skipping, it’s much more common for the Late ASL group in particular, given caregivers are also learning ASL and often not fluent. Prior work shows the proportion of produced words on a subset vs. full CDI are tightly correlated (R = 0.98) (Caselli et al., 2020).^6^ Notably, we re-ran the analyses using words that met our criteria for the Lancaster Sensorimotor Norms (without any additional hand-selection afterwards). I.e., we included all 16 visual, 8 auditory, and 13 abstract words. We observed the same pattern of results reported below; our data and code are on OSF, should curious readers seek to explore other categorizations.^7^ While using frequency *ratings* differs methodologically from the corpus-based frequency measures for English, subjective frequency ratings and corpus-derived frequency estimates tend to be highly correlated (e.g., Brysbaert & Cortese, 2011; Chen & Dong, 2019).
